# Metabolic Profiling of Endophytic Bacteria in Relation to Their Potential Application as Components of Multi-Task Biopreparations

**DOI:** 10.1007/s00248-023-02260-4

**Published:** 2023-07-01

**Authors:** Małgorzata Woźniak, Renata Tyśkiewicz, Sylwia Siebielec, Anna Gałązka, Jolanta Jaroszuk-Ściseł

**Affiliations:** 1 Department of Agricultural Microbiology, Institute of Soil Science and Plant Cultivation–State Research Institute, Czartoryskich 8, 24-100 Pulawy, Poland; 2 Analytical Laboratory, Łukasiewicz Research Network–New Chemical Syntheses Institute, Al. Tysiąclecia Państwa Polskiego 13a, 24-110 Pulawy, Poland; 3 Department of Industrial and Environmental Microbiology, Faculty of Biology and Biotechnology, Maria Curie-Skłodowska University, Akademicka 19, 20-033 Lublin, Poland

**Keywords:** Biotic and abiotic stress, Endophytic bacteria, Enhance plant stress resistance, Plant protection, Metabolic activity

## Abstract

**Abstract:**

Agricultural crops are exposed to various abiotic and biotic stresses that can constrain crop productivity. Focusing on a limited subset of key groups of organisms has the potential to facilitate the monitoring of the functions of human-managed ecosystems. Endophytic bacteria can enhance plant stress resistance and can help plants to cope with the negative impacts of stress factors through the induction of different mechanisms, influencing plant biochemistry and physiology. In this study, we characterise endophytic bacteria isolated from different plants based on their metabolic activity and ability to synthesise 1-aminocyclopropane-1-carboxylic acid deaminase (ACCD), the activity of hydrolytic exoenzymes, the total phenolic compounds (TPC) and iron-complexing compounds (ICC). Test GEN III MicroPlate indicated that the evaluated endophytes are highly metabolically active, and the best used substrates were amino acids, which may be important in selecting potential carrier components for bacteria in biopreparations. The ACCD activity of strain ES2 (*Stenotrophomonas maltophilia*) was the highest, whereas that of strain ZR5 (*Delftia acidovorans*) was the lowest. Overall, the obtained results indicated that ∼91.3% of the isolates were capable of producing at least one of the four hydrolytic enzymes. In addition, most of the tested strains produced ICC and TPC, which play a significant role in reducing stress in plants. The results of this study suggest that the tested endophytic bacterial strains can potentially be used to mitigate climate change-associated stresses in plants and to inhibit plant pathogens.

**Graphical Abstract:**

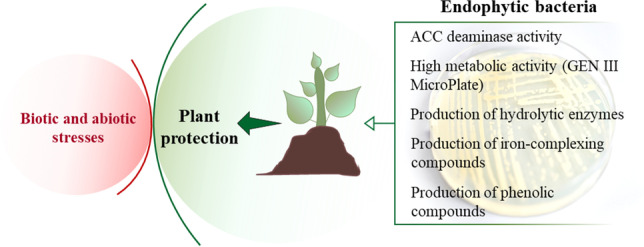

## Introduction

Agriculture plays a key role within the world economy. Agricultural production is a result of complex interactions amongst plants, soil, water, biodiversity, fertilisers, and agrochemicals. For the proper functioning of this complex network, all components need to be managed adequately [1]. The Food and Agriculture Organization Director-General Qu Dongyu emphasises that “Plants are the core basis of life on Earth” and “the most important pillar of human nutrition” and that “healthy plants are not something that we can take for granted”. Plants account for 80% of the food consumed and are of critical significance to ensuring food security and continued access to sufficient, affordable, safe, and nutritious food [2]. The life cycle of plants (growth and development phase) is sensitive to environmental conditions. Crops are grown under constantly changing environmental conditions, with potential adverse impacts on their growth and development. These negative impacts on plants are determined as crop stress. Agricultural stress has been identified as external factors that can cause irregularities in the crop cycle [3].

Two types of environmental stresses are classified, namely (1) abiotic and (2) biotic stress. Abiotic stress is caused by salinity, drought, flooding, cold and heat, heavy metals, and nutrient deficiency, amongst others, whereas biotic stress is a result of various organisms such as viruses, bacteria, fungi, nematodes, insects, herbivores, and weeds [1]. Biotic and abiotic stress factors are global issues that cause substantial economic losses and threaten food security. In addition, multidimensional stresses have a profoundly negative impact on morphological, physiological, and biochemical parameters as well as molecular processes in plants [4]. Savary et al. [5] indicated that pathogens, insects, pests, and weeds are responsible for yield losses ranging from 17.2% in potato up to 30.0% in rice. Another study reported crop yield losses of approximately 51–82% crop yield due to major abiotic stress factors [3].

Global climate change is a problem of high priority as it increases the frequency of extreme weather events and influences crop production worldwide. In addition to the direct effects of abiotic factors associated with changing climatic conditions, climate change can also increase the number of pests and weeds and the incidence of disease [3]. Climate change may facilitate plant infection in many ways: by changing pathogen evolution, altering host-pathogen interactions and vector physiology, and facilitating the emergence of new strains of pathogens that in turn can break host plant resistance [6]. Increased temperature can cause the development of new strains of pathogens and increase their abundance that are better adapted and more virulent. Moreover, it is anticipated that global warming is projected to increase development and movement of plant pests, which can affect their population dynamics by influencing fecundity, survival, generation time, population size, and geographic distribution [7]. The impacts of drought on infection rates of pathogens and disease severity are very different. For example, an increase in the length and frequency of drought can cause diseases, such as pea root rot (caused by *Aphanomyces euteiches*), onion white rot (*Sclerotium cepivorum*), wheat take-all (*Gaeumannomyces graminis* var. *tritici*), and wheat crown rot (*Fusarium* spp.) [6]. Moreover, dry climates may provide suitable conditions for the development and growth of herbivorous insects, and drought-stressed plants attract some insect species. Plants stressed by drought are more susceptible to insect attack because of a decrease in the production of secondary metabolites that have a defence function [8].

One of the possible alternatives to reducing the different abiotic and biotic stresses is beneficial microorganisms that interact with plants [9]. Microorganisms, an important component of global ecosystems are integral members of crop production systems and key elements of sustainable management in agriculture and agroecosystems [10]. The crop microbiome plays important roles in plant performance, nutrient acquisition, resistance against diseases, and tolerance to abiotic stresses [11]. The metabolic activity and specific genetic properties of bacterial endophytes make them suitable microorganisms to mitigate extreme environmental conditions. Endophytic interaction between plants and bacteria through various synergistic and defence mechanisms can increase the metabolic capacity of plants to cope with environmental stresses [12].

The main mechanism used by bacteria to limit stress in plants is the reduction of ethylene levels through the hydrolysis of 1-aminocyclopropane-1-carboxylic acid (ACC), the precursor of ethylene in all higher plants, catalysed by the enzyme ACC deaminase (ACCD) [13]. Ethylene is involved in plant responses to various biotic and abiotic stresses; however, high levels of ethylene can inhibit root elongation and growth, leading to plant deterioration [14]. Thus, the enzymatic activity of ACC deaminase prevents excessive increases in the synthesis of ethylene under various stress conditions, making it one of the most efficient mechanisms to induce plant tolerance [15].

Another mechanism used by bacteria for the direct protection of plants is the production of extracellular hydrolytic enzymes that attack the structural components of the cell walls of most fungi and play an important role in fungal mycelium degradation [16, 17]. Many bacteria are capable of synthesising extracellular enzymes that hydrolyse the variety of polymeric compounds, such us chitin, proteins, cellulose, hemicellulose, and DNA of phytopathogens. These hydrolytic enzymes affect the structural integrity of the cell wall of the targeted pathogens. For example, proteases show a key role in the lysis of cell wall of phytopathogenic fungi, because chitin and/or fibrils of β-glucan (major constituents of the cell walls) are embedded into the protein matrix. The protease enzyme breaks down major proteins of phytopathogenes into peptide chains and/or amino acids and thereby destroys their capacity of pathogen’s protein to act on plant cells. Cellulases hydrolyse the 1,4-β-D-glucosidic linkages in cellulose, one of the main components of the cell walls of fungi and pathogenic oomycetes [18, 19].

Iron (Fe) is one of the essential trace elements involved in plant metabolism, and Fe deficiency can lead to abnormal respiration and photosynthesis. In soils, the total Fe content often exceeds the requirements of plants, but its bioavailability is insufficient under various environmental conditions (iron in the form of Fe^3+^ forms insoluble hydroxides and oxyhydroxides). Plants and microorganisms have therefore developed active strategies for iron uptake. Bacteria obtain iron by secreting iron-compensating compounds. These bacteria play a particularly important role in an environment where the plant is exposed to iron deficiency. In addition, iron-complexing compounds mediate biological control mechanisms against phytopathogens, limiting their iron availability due to nutritional competition and, thus, inhibiting their proliferation and plant colonisation [20, 21].

Plants produce phenolic compounds mainly as a result of stressful external factors. Phenolics play a role in plant defence mechanisms to pathogens and major abiotic stresses such as drought, salinity, and UV [22]. However, microorganisms also produce phenol. For example, *Debaryomyces hansenii* can convert ferulic acid into several types of phenol compounds, using glucose and nitrogen [23]. Endophytic fungi biosynthesize phenolic compounds via the shikimate pathway [24]. It is therefore suggested that biofertilisation with microorganisms can reduce the negative impact of stress factors on plants as a result of the potential production and biosynthesis of several phenolic compounds [25].

Against the background of a rapidly changing climate, the production of crops than can adapt to new environmental conditions is becoming increasingly important. In this context, the aim of this study was to characterise native strains of endophytic bacteria from several crops in terms of features contributing to increasing plant resistance to biotic and abiotic stresses. The present study was designed to analyse the properties (production of ACC deaminase, hydrolytic exoenzymes, phenolic compounds, and iron-complexing compounds) that are involved in increasing plant tolerance to stress.

## Materials and Methods

### Endophytic Bacteria

The bacterial strains used in this study were obtained from the bacterial collection of the Department of Agricultural Microbiology, Institute of Soil Science and Plant Cultivation, State Research Institute, Pulawy, Poland. Overall, 23 strains of endophytic bacteria were selected from the tissues of four different crops and two wild plants (Table 1). Bacteria were isolated from healthy and mature plants: *Zea mays* L. (maize), *Vicia faba* L. (broad bean), *Secale cereale* L. (rye), *Triticum aestivum* L. (wheat), *Arctium lappa* L. (burdock), and *Equisetum arvense* L. (horsetail). A description of the isolation process and the results of their identification based on 16S rRNA gene sequencing are presented by Woźniak et al. [10]. The biochemical characteristics of these strains are included by Woźniak et al. [26].

**Table 1:** List of endophytic bacterial strains used in this study

Strain	Host plant	Identification	GenBank accession no.
ZR1	*Zea mays*	*Novosphingobium resinovorum*	KY486807
ZR3		*Delftia acidovorans*	KY486832
ZR4		*Delftia acidovorans*	KY486833
ZR5		*Stenotrophomonas* sp*.*	KY486808
ZS2		*Delftia acidovorans*	KY486834
ZS5		*Delftia acidovorans*	KY486835
ZS6		*Delftia* sp*.*	KY486831
VR2	*Vicia faba*	*Variovorax paradoxus*	KY486805
VS3		*Rhizobium* sp*.*	KY486825
VS4		*Delftia acidovorans*	KY486829
SR1	*Secale cereale*	*Delftia* sp*.*	KY486822
SR3		*Delftia acidovorans*	KY486810
SS5		*Delftia acidovorans*	KY486813
TS1	*Triticum aestivum*	*Delftia acidovorans*	KY486817
TS4		*Delftia acidovorans*	KY486820
AR2	*Arctium lappa*	*Collimonas pratensis*	KY486811
AR3		*Achromobacter xylosoxidans*	KY486824
AR4		*Stenotrophomonas maltophilia*	KY486847
ER1	*Equisetum arvense*	*Comamonas koreensis*	KY486814
ES1		*Rhizobium* sp.	KY486815
ES2		*Stenotrophomonas maltophilia*	KY486848
ES4		*Brevundimonas* sp*.*	KY486828
ES7		*Brevundimonas* sp*.*	KY486816

### Phenotypic Profiling of Endophytic Bacteria using Biolog™ GEN III MicroPlates

Pure cultures of the endophytic bacteria were characterised by the Biolog GEN III system (Biolog Inc. Hayward, CA, USA), following the manufacturer’s instructions. This method allows the establishment of a metabolic profile for specific microorganisms, i.e., a “phenotypic fingerprint”.

### Qualitative Estimation of ACC Deaminase Production by Bacterial Endophytes

Qualitative estimation was performed via the method described by Govindasamy et al. [27]. To determine the ACC deaminase activity, bacterial strains were grown with three different nitrogen sources: (A) positive control—DF (Dworkin Foster) (minimal medium [28] + (NH_4_)_2_SO_4_ (0.2% w/v); (B) negative control—DF minimal medium (without nitrogen source); (C) DF + 3mM ACC (Sigma-Aldrich, USA) (as a sole nitrogen source). The plates were incubated at 28 °C for 72 h. The growth of bacteria on plates with the addiction of ACC was compared with that of the positive and negative controls. Growth on ACC (C) medium indicates the ability of the tested strains to use ACC as the sole nitrogen source and synthesise the enzyme ACC deaminase [29, 30].

### ACC Deaminase Activity Assay

The quantitative analysis of ACC deaminase activity was performed according to a modified method of Penrose and Glick [29], which is based on measuring the amount of α-ketobutyrate (α-KB) produced during ACC hydrolysis. The endophytic bacterial cells were grown at 28 °C in TSB medium (Tryptic Soy *Broth, Sigma-Aldrich, USA).* After overnight growth, the bacterial cultures were centrifuged at 10,000 × g for 10 min, washed with 0.1 M Tris–HCl (pH 7.5), and suspended in 5 mL of DF medium containing 3 mM ACC as the sole nitrogen source. The cultures were incubated for 48 h at 28 °C, and the biomass was collected by centrifugation at 10,000 × g for 10 min at 4 °C. The supernatant containing the cells was removed, and the cells were washed twice with 5 mL of DF salt minimal medium. The cells were then resuspended in a fresh culture tube in 7.5 mL of DF salt minimal medium supplemented with 3 mM ACC and incubated at 28 °C with shaking for a further 24 h (140 rpm/min.). The induced cells were then harvested by centrifugation at 6000 × g for 10 min and washed with 0.1 M Tris-HCl (pH 7.6). The contents of the tubes were centrifuged at 12,000 × g for 5 min, and the pellets were resuspended in 200 μL of 0.1 mol L^−1^ Tris HCl (pH 8.5). After washing, the bacterial cells were labilised by adding 10 μL of toluene and then vortexed at the highest speed for 30 s. Subsequently, 50 μL of toluenised cell suspension was placed in a fresh tube, and 5 μL of 0.5 M ACC was added, followed by shaking and incubating at 30 °C for 15 min. A 250-μL aliquot of the supernatant was transferred to a glass tube and spiked with 200 μL of 0.56 N HCl and 75 μL of 2,4-DNF (0.2% 2, 4-dinitrophenylhydrazine in 2 mol L^−1^ HCl) solution, and the contents were mixed and incubated at 30 °C for 30 min. Next, 500 μL of NaOH (2N) was added, and the absorbance of the obtained mixture was measured at 540 nm by using a Varian Cary 1E UV-visible spectrophotometer. The cell suspension without ACC was used as a negative control and that with (NH_4_)_2_SO_4_ as a positive control. The values of the concentration of α-ketobutyrate produced were determined by constructing a standard curve ranging from 0.1 to 1 mM. The ACC deaminase activity was calculated as the amount of α-KB produced in μmol mg^−1^ protein h^−1^. The total protein content was estimated using the Bradford method (BioRad, Hercules, CA, USA) according to the manufacturer’s protocol [31].

### Screening Isolates for Extracellular Hydrolytic Enzyme Activity

The selected bacteria were screened for extracellular hydrolytic activity of protease, cellulase, lipase, and esterase in agar plate assays. For the assessment of protease activity, one loop full of endophytic bacterial strains was streaked on a skimmed milk agar plate containing 5% (w/v) skim milk (Sigma-Aldrich, USA). Proteolytic activity was observed by the development of a halo zone around the colony of endophytic bacterial isolates [16]. For determining cellulolytic activity, bacterial strains were spot-inoculated on agar plates with carboxy methyl cellulose (CMC) (Sigma-Aldrich, USA), using the method described by Meddeb-Mouelhi et al. [32]. Screening of strains for lipolytic activity was performed according to Ramnath et al. [33]. Activated cultures of each endophyte were streaked on agar media containing suitable substrate specific for each of the activities esterase and lipase, respectively, namely Tween 20 and Tween 80 (Sigma-Aldrich, USA). The estimated sizes of the decolonisation zones (halos) were used as a measure of the enzymatic activity (Enzyme index = EI) in all tests.

### Determination of the Total Phenolic Compounds

The concentration of total phenolic compounds (TPC) was determined in the supernatants of bacterial strains cultured on TSB medium for 48 h at 28 °C. The concentration of TPC was determined by reaction with Folin-Ciocalteu reagent [34]. Spectrophotometric measurement (Varian Cary 1E UV-visible) was performed, determining the absorbance value at λ = 680 nm. The concentration of TPC was calculated based on the determined calibration factor after preparing the standard curve for standard solutions of ferulic acid (US Pharmacopeia, USA).

### Determination of the Total Content of Iron-Complexing Compounds

The concentration of the total content of iron (Fe^3+^)-complexing compounds (ICC) was determined in the supernatants of bacterial strains cultured on TSB medium for 48 h at 28°C. For this, 300 μL of 6% FeCl_3_ × 6H_2_O in 1 N HCl was added to 1 mL of the obtained supernatants, and the samples were thoroughly mixed and incubated for 1 h at room temperature. Subsequently, spectrophotometric measurements (Varian Cary 1E UV-visible) were performed, determining the absorbance value at λ = 520 nm [35]. The concentration of ICC was calculated based on the determined calibration factor after preparing the standard curve for standard solutions of desferrioxamine methanesulfonate B (DFOB, Sigma-Aldrich, USA)

### Statistical Analysis

All experiments were performed in triplicate, and the data are presented as means ± standard deviation (SD). The data from Biolog GEN III experiments were combined in a single matrix, represented as a positive integer, OmniLog TM units (OL units) and subjected to statistical analysis using the STATISTICA.PL (13.1) software (StatSoft Inc., Tulsa, OK, USA) for presentation as a heat map graph. A multivariate statistical method using PCA was performed to summarise the variability of the tested strains and determine the association amongst the measured activities. Mean values from three replicates were used for PCA analysis. Prior to this analysis, the data were standardised.

## Results

### Biolog GEN III-Based Metabolic Assay

**Fig. 1:**
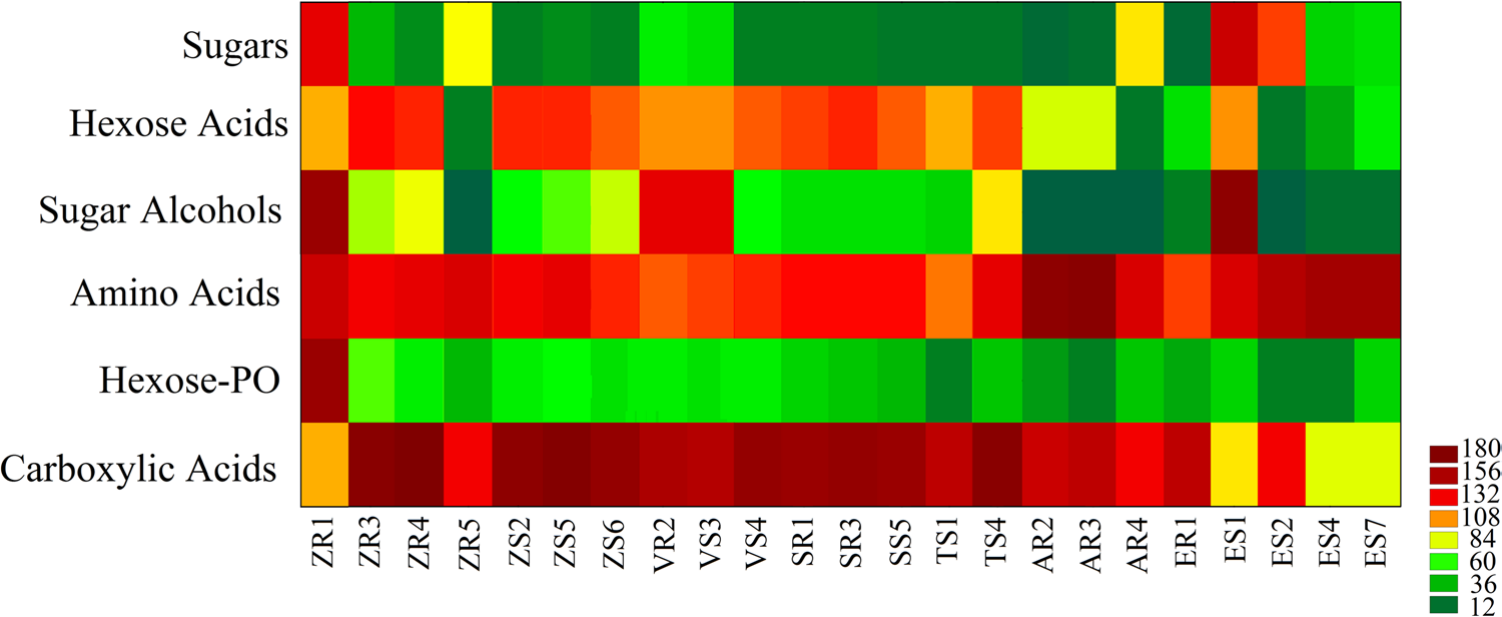
Microbial use of the selected carbon substrates by endophytic bacteria

To investigate the overall metabolic profiles of endophytic bacteria, the substrates on the GEN III MicroPlate were categorised into six classes: sugars, sugar alcohols, hexose-PO, amino acids, hexose acids, and carboxylic acids. The results of the relative use of the chemical groups of subtrees are presented in Fig. 1. Generally, regardless of the strains, amino acids and carboxylic acids were used as the most used carbon substrates (Fig. 1).

### Screening for ACC Deaminase Activity

All analysed strains showed the ability to grow on solid medium with ACC, albeit at different intensities (Table 2). Based on the obtained results, all isolates were positive for the activity of ACC deaminase, but strains ZS2, VS4, TS4, and AR2 showed the lowest ability to grow on DF medium with ACC. Not all isolates growing on DF minimal medium with 0.2% ammonium sulphate were also able to grow on nitrogen-free media. Therefore, to confirm their ability to produce deaminase ACC, quantitate analysis was done based on the spectrophotometric method.

**Table 2:** Screening of the growth intensity of endophytic bacteria on DF media supplemented with ammonium sulphate, without any nitrogen source and ACC as nitrogen source

Strain	Growth on DF medium supplemented with		
	Medium with (NH_4_)_2_ SO_4_ (positive control)	Medium without any nitrogen source (negative control)	Medium with ACC as sole nitrogen source
ZR1	+++	++	++
ZR3	+++	+	++
ZR4	+++	+	++
ZR5	+++	+	+
ZS2	+++	+	++
ZS5	+++	+	++
ZS6	+++	++	++
VR2	+++	+	++
VS3	+++	+	++
VS4	+++	−	+
SR1	+++	+	+
SR3	+++	++	++
SS5	+++	++	++
TS1	+++	+	++
TS4	+++	+/−	+
AR2	+++	+	++
AR3	+++	++	+
AR4	+++	+	++
ER1	+++	+	++
ES1	+++	+	++
ES2	+++	+	+++
ES4	+++	+	++
ES7	+++	++	++

Degree of intensity of growth: +++, high; ++, medium; +, low; −, lacking

### ACC Deaminase Activity

**Fig. 2:**
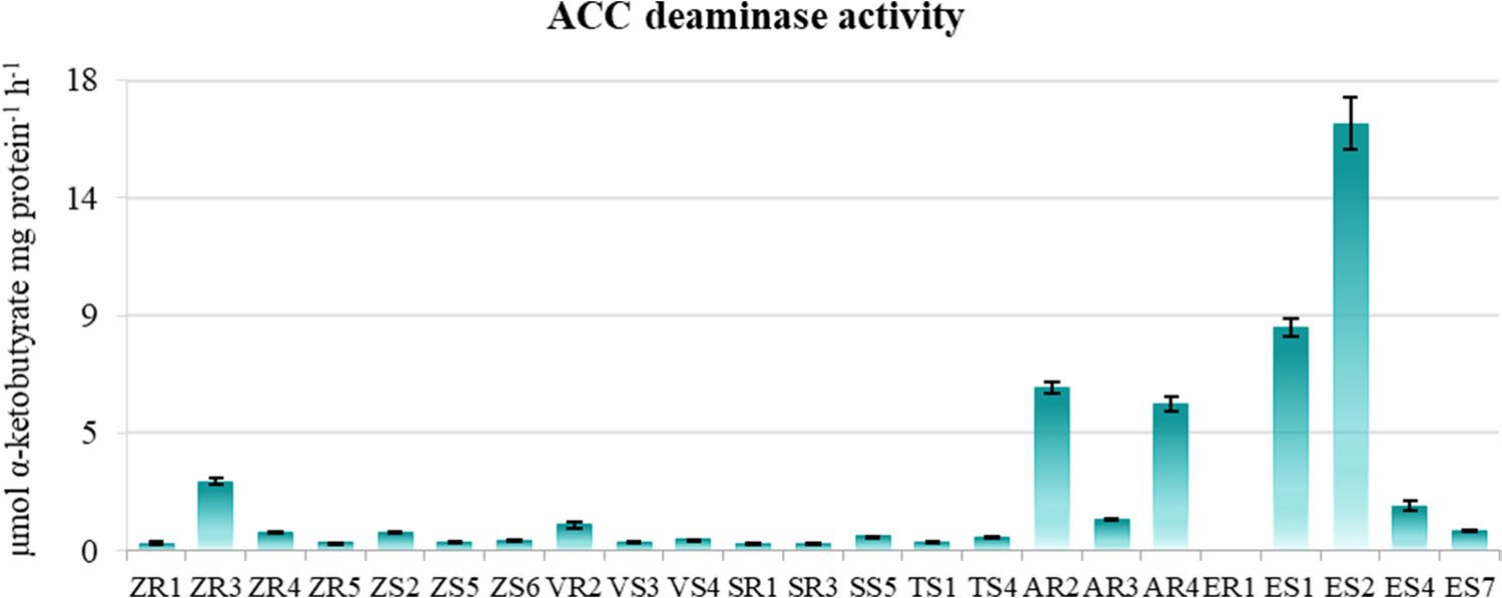
ACC deaminase activity values (μmol α-ketobutyrate mg protein^−1^ h^−1^) of the endophytic bacteria tested in minimal medium with ACC. Bars indicate standard deviation (SD)

Amongst the 23 endophytic bacterial isolates, 22 strains (96%) showed variation in ACC deaminase activity in the range of 0.264–16.138 μmol α-KB mg protein^−1^ h^−1^ in DF minimal salt broth medium supplemented with ACC as nitrogen source. Only strain ER1 showed no ability to synthesise ACC deaminase. Moreover, strain ER1 showed the ability to grow on DF medium supplemented with ACC as a sole source of nitrogen, but it showed no ACC deaminase activity (Table 2 and Fig. 2). The highest ACC deaminase activity was observed for bacterial strain ES2 (16.138 μmol α-KB mg protein^−1^ h^−1^) followed by ES1 (8.511 μmol α-KB mg protein^−1^ h^−1^), AR2 (6.446 μmol α-KB mg protein^−1^ h^−1^), and AR4 (5.505 μmol α-KB mg protein^−1^ h^−1^). These strains of endophytic bacteria belonged to the genera *Stenotrophomonas* (ES1 and AR4), *Rhizobium* (ES1), and *Collimonas* (AR2). By contrast, the lowest level of ACC deaminase production was observed in isolates ZR5 and ZR1 (Fig. 2). In the medium without ACC, the ability to synthesise ACC deaminase was recorded for only four strains (16%), with the concentration of α-KB being as low as 0.009 to 0.081 μmol α-KB mg protein^−1^ h^−1^ (data not shown). Four of the tested isolates demonstrated the ability to produce ACC deaminase at different concentrations, both in medium with and without ACC. The addition of ACC as a nitrogen source significantly increased the efficiency of ACC deaminase biosynthesis.

### Screening Isolates for Extracellular Hydrolytic Enzyme Activity

All bacterial isolates were screened for protease, cellulase, lipase, and esterase activities (Table 3). Preliminary tests on media enriched with the substrate for each enzyme showed that 21 (∼91.3%) isolates expressed at least one of the four enzymes, whereas 2 (∼8.7%) strains showed none of the selected hydrolase enzyme activities; however, all different enzyme activities were detected in 8 (∼34.8%) isolates. Amongst the 23 endophytic bacterial isolates, 65.22%, 52.16%, 86.96, and 82.60% of the strains possessed protease, cellulase, lipase, and esterase activities, respectively. We also observed varied values of enzyme indices for all hydrolytic activities, suggesting that some isolates displayed higher activity than others. The highest protease activity was found for strain ZR4 (EI = 5.172), classified as *Delftia acidovorans*, whereas the highest cellulase activity was detected in *Delftia acidovorans* isolate SR3 (EI = 5.583). Strain TS4 showed the highest lipase synthesis activity, and strain TS1 showed the highest esterase activity; both strains were classified as *Delftia acidovorans*.

**Table 3:** Hydrolytic enzyme activity profiles of the endophytic bacteria expressed on the basis of the enzyme index

Strain	Extracellular hydrolytic enzyme activity			
	Protease (P)	Cellulase (C)	Lipase (L)	Esterase (E)
ZR1	0.000	5.439 ± 0.134	0.000	0.000
ZR3	4.368 ± 0.039	0.000	10.089 ± 0.646	2.955 ± 0.034
ZR4	5.172 ± 0.045	3.190 ± 0.038	5.549 ± 0.389	3.018 ± 0.067
ZR5	4.067 ± 0.030	5.294 ± 0.133	4.737 ± 0.007	3.751 ± 0.022
ZS2	3.163 ± 0.023	0.000	5.656 ± 0.616	3.618 ± 0.051
ZS5	3.332 ± 0.037	0.000	6.491 ± 0.576	3.788 ± 0.071
ZS6	3.002 ± 0.002	0.000	4.064 ± 0.085	4.358 ± 0.190
VR2	0.000	3.884 ± 0.154	7.614 ± 0.470	5.225 ± 0.154
VS3	0.000	0.000	4.791 ± 0.083	3.180 ± 0.025
VS4	3.197 ± 0.010	5.107 ± 0.026	7.948 ± 0.271	2.904 ± 0.067
SR1	3.256 ± 0.030	4.765 ± 0.086	5.698 ± 0.359	4.543 ± 0.039
SR3	2.791 ± 0.022	5.583 ± 0.109	5.043 ± 0.336	3.425 ± 0.069
SS5	3.244 ± 0.011	0.000	7.605 ± 0.328	3.266 ± 0.058
TS1	3.848 ± 0.008	5.453 ± 0.033	3.152 ± 0.100	8.222 ± 0.414
TS4	2.667 ± 0.008	5.530 ± 0.080	11.664 ± 0.886	2.850 ± 0.026
AR2	0.000	0.000	0.000	0.000
AR3	0.000	0.000	0.000	0.000
AR4	3.884 ± 0.068	0.000	3.932 ± 0.017	3.396 ± 0.037
ER1	0.000	0.000	5.966 ± 0.461	4.765 ± 0.174
ES1	0.000	5.464 ± 0.069	5.155 ± 0.028	0.000
ES2	4.494 ± 0.021	3.468 ± 0.046	3.505 ± 0.081	2.481 ± 0.096
ES4	0.000	5.112 ± 0.063	2.426 ± 0.021	2.441 ± 0.009
ES7	2.511 ± 0.023	0.000	4.027 ± 0.058	3.677 ± 0.256

### Evaluation of the Total Contents of Iron-Complexing and Phenolic Compounds

**Fig. 3:**
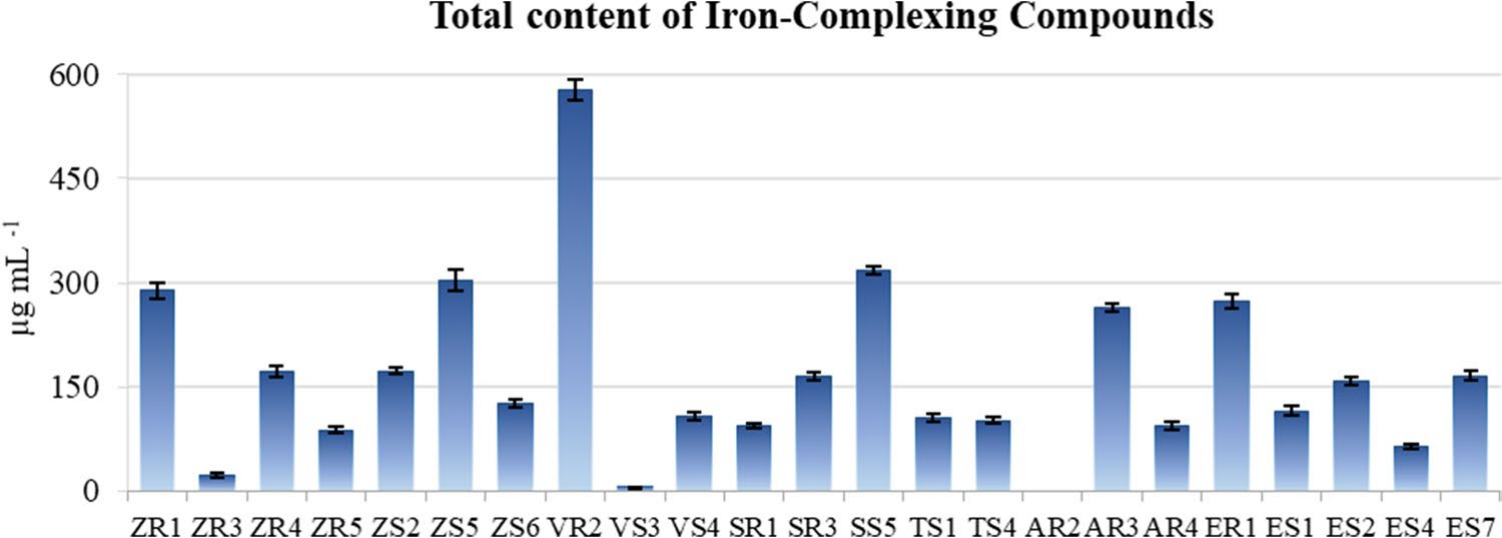
Contents of iron-complexing compounds (ICC) in the cultures of the tested endophytic bacterial strains. Bars indicate standard deviation (SD)

**Fig. 4:**
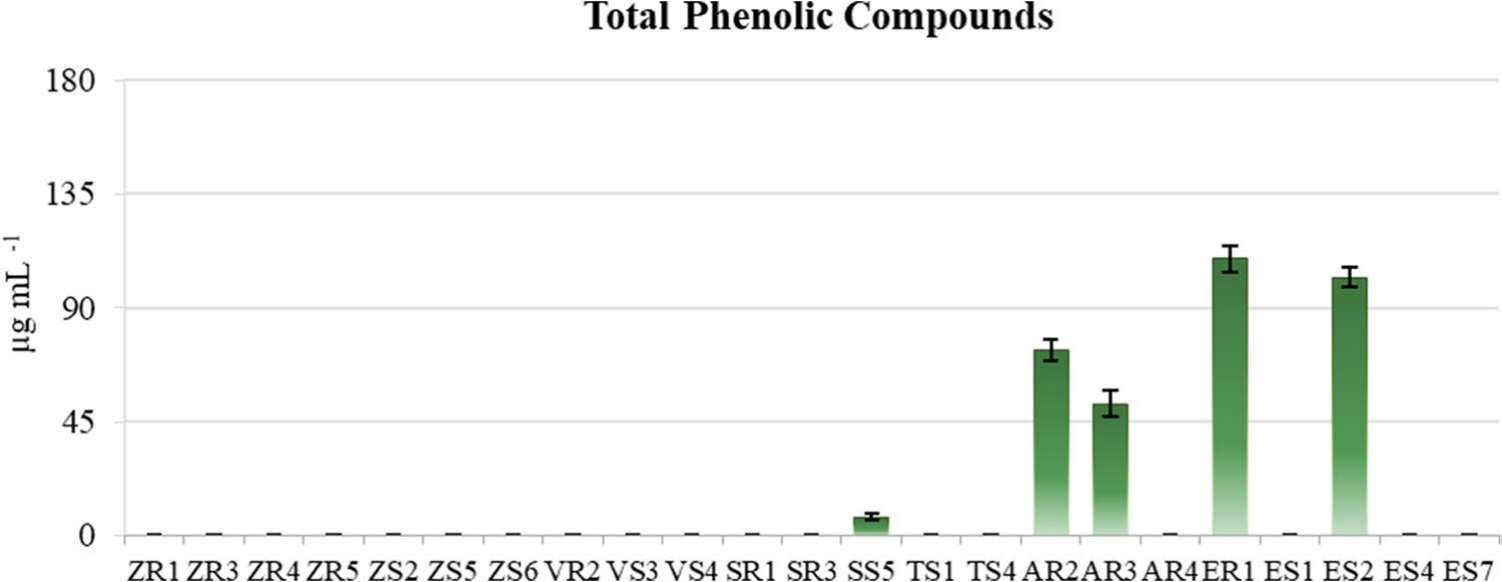
Contents of total phenolic compounds (TPC) in the cultures of the tested endophytic bacterial strains. Bars indicate standard deviation (SD)

Quantitative analysis of the total contents of iron-complexing compounds (ICC) showed that almost all tested strains produced these compounds. Only strain AR2 showed no ability to produce ICC, whereas strains ZR3 and VS3 had the lowest concentrations of these compounds. The highest ICC level was found in the culture of the *Variovorax paradoxus* strain VR2 (576.516 μg mL^−1^). However, the contents of these compounds in the cultures of the other tested strains ranged from 6.015 to 316.779 μg mL^−1^ (Fig. 3). Of the 23 tested strains, 5 (∼21.7%) showed the ability to produce phenolic compounds (TPC). The content of TPC in the cultures of these strains (SS5, AR2, AR3, ER1, ES2) ranged from 7.901 to 109.592 μg mL^−1^. In addition, the TPC content was the lowest in the culture of the *Delftia acidovorans* strain SS5 and the highest in the culture of the *Stenotrophomonas maltophilia* strain ES2 (Fig. 4).

### Principal Component Analysis

**Fig. 5:**
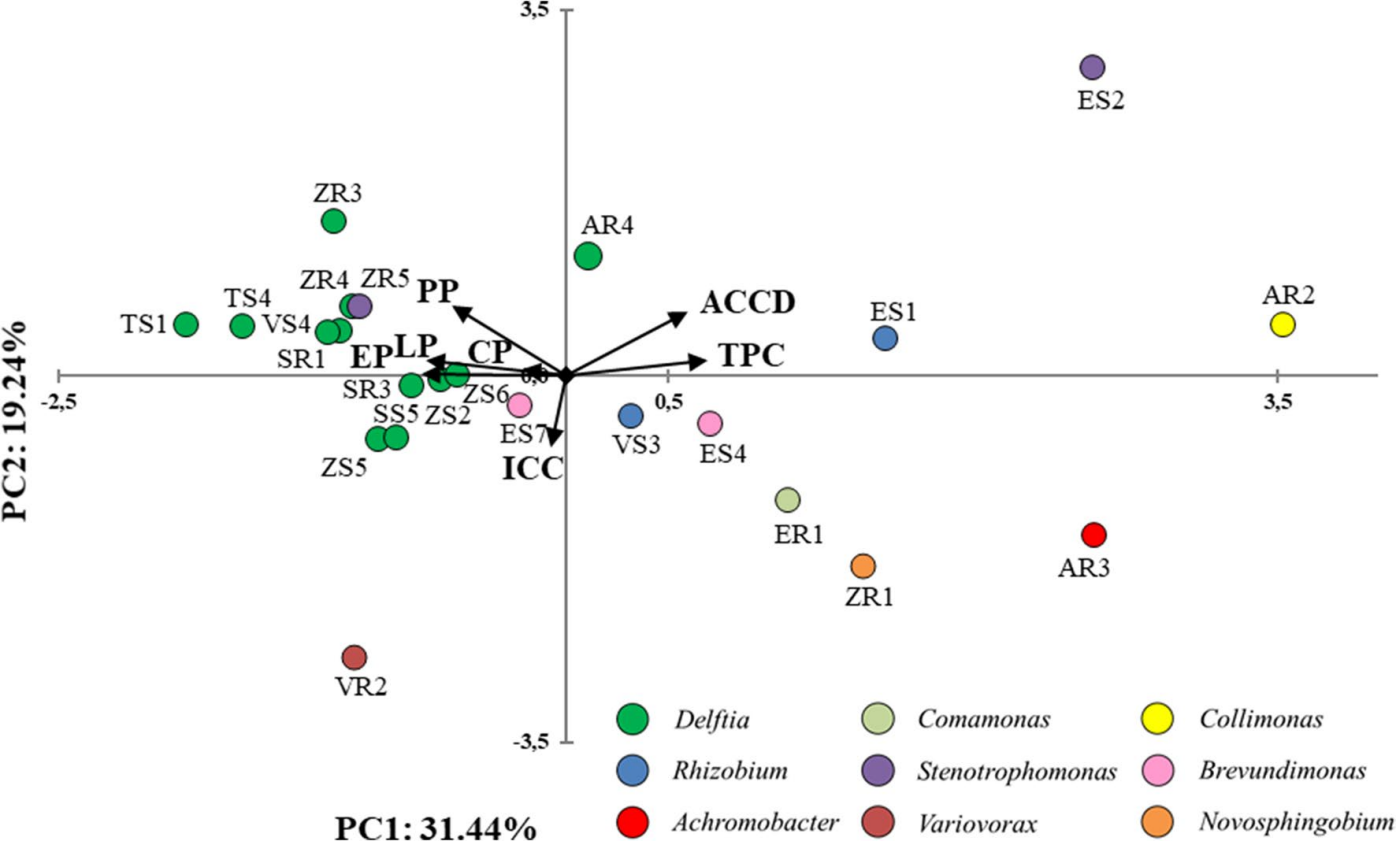
Biplot diagram of principal component analysis (PCA), describing the activities of 23 bacterial endophytes isolated from different plants. Strains classified based on the nucleotide sequence of their 16S rRNA genes to the same genus are indicated in the same colours. Abbreviations: ACCD, ACC deaminase activity; TPC, total phenolic compounds; ICC, iron-complexing compounds; P, protease activity; C, cellulase activity; L, lipase activity; E, esterase activity

The relationships amongst all endophytic bacteria and their properties were assessed via principal component analysis (PCA) (Fig. 5). The first two principal components (PCs) accounted for 31.44% and 19.24% of the total variance of the tested bacterial isolates. The extracellular hydrolytic enzyme activity (PP, CP, LP, and EP) was positively related for strains from the genus *Delftia* and one strain from the genus *Stenotrophomonas*, isolated from crop plants. In contrast, strains collected from weeds were positively related to the ability to produce deaminase ACC and phenolic compounds. The PCA showed a separation of the evaluated strains based on their genus, resolving strains based on taxonomic affiliation and plant type (crop plants and weeds).

## Discussion

In the context of the ecological and climate crises we are currently facing, along with the growing demand for food and the limitation of natural resources, agriculture must undergo a transformation towards maintaining balance and natural stability, biodiversity, and soil health [1]. Increasing plant resistance through the use of microbial preparations to mitigate climate change-associated stresses is a sustainable way to ensure food security and reduce the use of chemicals in agriculture [12].

With an increasing awareness of sustainable farming practices, it is important to seek region-specific microbial strains (native strains) that have a broad spectrum of activity and are compatible with other microorganisms in soil and plant tissues for the use as components of inocula to enhance plant resistance [36]. The findings presented in Woźniak et al. [26] on the evaluation of the same 23 strains of endophytic bacteria, based on the characterisation of direct mechanisms affecting plant growth and development, are promising and form the basis of this study on the indirect mechanisms affecting plant protection. Fikri et al. [37] showed that locally isolated endophytic bacteria are more effective against local pathogens than bacteria from different parts of the world. Another study found that isolates of native root bacteria, introduced to cultivation de novo, e.g., as biopreparations, can effectively support plant growth [11]. Most research on microbial components of biopreparations focused on the analysis of rhizosphere bacteria [38]. In our study, we analysed previously studied and active PGPE—plant growth-promoting endophytes [26]. Bacterial endophytes may have an advantage over rhizosphere-dwelling bacteria because living in plant tissues offers the possibility of constant direct “contact” with plant cells, facilitating a direct beneficial effect in exchange for a constant supply of nutrients [39].

The Biolog GEN III MicroPlate test is based on phenotypic tests and allowed the assessment of the metabolic profile of selected strains of endophytic bacteria. Analysis of the metabolic activity provides important information about individual environmental isolates, which is crucial in understanding their functioning in future environmental niches, carriers, and biopreparations. The wider the spectrum of the compounds used, the greater the ability of the strains to survive under different conditions and compete with other potentially pathogenic microorganisms [40]. In addition, the results of the Biolog GEN III test can be the basis for the selection of the carrier and additives for bacteria in biopreparations. Carefully selected chemical compounds are a valuable supplement to biomodifiers and can extend the shelf life and survival of the beneficial strain, e.g., in non-scaling stressful conditions [41]. In this study, the most intensively metabolised compounds were amino acids. These compounds, secreted from plant roots, are the basic source of carbon and nitrogen for bacteria, and most of the analysed strains actively metabolised amino acids. These compounds, as an addition to the carrier of bacteria in biopreparations, are not only a source of energy for bacteria but can also affect plant metabolism. In a previous study, the exogenous use of L-histidine increased resistance to pathogens, e.g., in *Solanum lycopersicum* and *Arabidopsis thaliana* [42]. On the other hand, L-aspartic acid is a metabolite necessary for the growth and development of plants and for plant defence responses to stress factors [43].

The ability of bacteria to synthesise ACC deaminase is one of the key growth-stimulating features affecting stress tolerance in plants under both normal and stress conditions [15]. According to Onofre-Lemus et al. [44], the bacterial enzyme ACC deaminase has a positive effect on plant physiology, growth, and development by reducing ethylene levels in plants. Therefore, the presence of microbial producers of ACC deaminase in biopreparations is extremely important in today’s agricultural system, which is more susceptible to climate change. In our study, we characterised 23 endophytic bacteria from tissues of four different crops and two wild plants. All isolates showed the ability to grow on DF minimal medium and on medium supplemented with ACC as a sole nitrogen source. Most of the bacterial strains were also able to grow on medium without any nitrogen source, indicating their capacity to fix atmospheric nitrogen [45]. These features were confirmed by amplification of the *nif*H gene in our previous study [26].

In the present study, the most active producers of ACC deaminase were strains of the genera *Stenotrophomonas*, *Rhizobium*, *Collimonas*, and *Delftia*. The indicated isolates showed high ACCD activity with > 5 μmol of α-KB mg protein^−1^ h^−1^; according to Singh and Jha [46], the production of > 20 nmol of α-KB mg protein^1^ h^1^ is sufficient to trigger systemic tolerance under stress conditions. The genus *Stenotrophomonas* has frequently been described as a genus with great potential in agriculture [47]. For instance, *Stenotrophomonas maltophilia* SBP-9 augments resistance against biotic and abiotic stress in wheat plants through, amongst others, synthetising ACCD at the level 362 nmol of α-KB mg protein^−1^·h^−1^ [46].

Belimov et al. [48] demonstrated that ACC deaminase activity in *Rhizobium leguminosarum* contributes to an increased efficiency of symbiosis with *Pisum sativum* L., both under single and complex stress conditions related to cadmium and water deficiency. Other authors demonstrated ACC deaminase activity in members of the genera *Collimonas* [49] and *Delftia* [50].

Interestingly, the most effective producers of ACC deaminase were isolated from tissues of the weeds *Arctium lappa* and *Equisetum arvense*, which are ubiquitous under various environmental conditions. In parallel with the crops, they grow and reproduce aggressively [51]. Like all plants, weeds can have a rich microbiota that can contribute to their survival in a wide range of environmental conditions. According to literature data, microorganisms play an important role in the ability of weeds to develop under suboptimal environmental conditions, contributing to plant resistance and other reinforcing functions [52]. In addition, weed-associated microorganisms can be transferred to cultivated plants, with great value in terms of their supply of beneficial functions [53].

In this study, bacterial endophytes were qualitatively analysed for their ability to produce different hydrolytic enzymes. Overall, microbial synthesis of the hydrolytic enzymes appears to be important for the colonisation of plant roots and biocontrol efficacy [16, 17]. In our study, the strains of the genera *Delftia* and *Stenotrophomonas* showed all tested hydrolytic enzyme activities. Wang et al. [54] emphasise that a wider range of hydrolytic enzymes produced by PGPB (plant growth-promoting bacteria) potentially provides these bacteria with an advantage in inhibiting many pathogens due to the complementary action of their lytic enzymes. To the best of our knowledge, this study is the first to provide a complex profile of four different extracellular hydrolytic enzymes produced by *Delftia acidovorans* strains isolated from different crop plants. According to the literature, strains of the *Stenotrophomonas* genus have a high hydrolytic potential to produce proteases, chitinases, glucanases, lipases, and other enzymes [55]. In particular, the proteolytic and chitinolytic activities of *S. maltophilia* contribute to protecting plants from fungal pathogens such as *Magnaporthe grisea* [56]. These endophytic bacteria might have an array of biological and environmental applications to control pathogens [17]. According to these authors, an enzymatic index (EI) above 1.0 indicates enzyme secretion outside the bacterial colony. Our study shows that all EI values of the evaluated bacterial strains for enzymes were above 1, proving their potential for biotechnological applications.

According to a previous study, amongst endophytic bacteria, the highest hydrolytic activity is recorded against protease and cellulase [57]. In contrast, in our study, we found a high number of endophytic strains with lipase and esterase activity. Moreover, the tested bacteria produced lipases and esterase to a greater extent compared to the other enzymes, which has not been previously reported. Probably, this relationship is due to the adaptation of these endophytic bacteria to the respective metabolic machinery of the host tissues and environmental conditions. The lipolytic activity of endophytic bacteria (production of lipase and esterase) is an extremely important feature because these enzymes are involved in the hydrolysis of the major components of the plant pathogen fungal cell walls [58]. In addition, bacteria that hydrolyse the lipids of the fungal cell wall can use the released fatty acids as a long-term source of energy [59]. Tayyrov et al. [60] suggested that the microbiological production of lipases can find applications in the control of parasitic nematodes in agriculture and medicine. Moreover, bacterial lipases can be used in the production of agrochemicals such as insecticides, fungicides, and herbicides, for example (S)-indanofan, which is used to control wild grasses and weeds [61].

The iron-complexing compounds are implicated directly by incentive nutrient uptake and indirectly by insulating Fe^3+^ in the areas around the roots, preventing its assimilation by pathogens and, thus, facilitating disease inhibition [20]. In our study, the *Variovorax paradoxus* (VR2) strain was the strongest producer of compounds capable of complexing iron. In a similar study, Liu et al. [62] proved that a *Variovorax boronicumulans* strain promoted plant growth by producing siderophores, which was also correlated with the production of other secondary metabolites (indole-3-acetic acid, IAA; salicylic acid, SA; hydrogen cyanide, HCN; ammonia, and solubilised phosphate) for biofertiliser and biopesticide applications. In addition to plant growth stimulation and biological control, iron-complexing compounds, including siderophores, are also used in bioremediation [21] as they are highly effective in solubilising and increasing the mobility of a wide range of metals that can be hazardous to the environment, e.g., Al, Cd, Cu, Ga, In, Pb, and Zn, as well as radioactive elements including U and Np [63, 64]. This process depends mainly on the functionality of the ligand, which means that siderophores may have a strong affinity for a specific metal other than iron [21]. Siderophores produced by *Agrobacterium radiobacter* can reduce the As content in the soil by approximately 54% [54].

Phenolic compounds comprise numerous groups of secondary metabolites with antibacterial and antioxidant properties. Built into the plant cell wall, they create structural barriers that prevent the development of the pathogen at the site of infection [65, 66]. Although phenols are mainly isolated from plants, they can also be produced by microorganisms [67]. Most literature data indicate the production of phenols by yeasts and fungi [23, 24]. However, information regarding bacteria is limited to a few reports. For example, amongst several compounds produced by the endophytic strain *Bacillus thuringiensis*, phenols are crucial in controlling root-knot nematodes [68]. It was also demonstrated that the inoculation of tomato seeds with endophytic bacteria of the genera *Bacillus*, *Pseudomonas*, and *Methylophilus* indirectly increased the total phenol content in tomato [69]. Interestingly, Akter et al. [70], based on an in silico approach, report that endophytic bacteria isolated from medicinal plants can provide new bioactive compounds, amongst other phenolic compounds, against target proteins of SARS-COV-2. Our research shows that endophytic bacterial strains, such as *C. pratensis* (AR2), *C. koreensis* (ER1), *A. xylosoxidans* (AR3), and *S. maltophilia* (ES2), are a rich source of phenolic compounds, which translates into their potential use in the direct and indirect protection of plants against biotic stresses.

The results obtained by the analysis of various properties of endophytic bacteria indicate that the complex features responsible for enhancing plant stress resistance are mainly determined by the type of plants and, partially, by the endophytic bacterium. Our previous studies based on PCA analysis clearly showed that the plant growth-promoting features of these endophytic bacteria were dependent on the bacterial genus, as identified by 16S rRNA gene sequencing [26]. Jacoby et al. [71] report that plants shape microbial communities most probably by root exudates. It is assumed that plants decide not only which microorganisms can colonise them but also what characteristics will be available to these microorganisms. The type of the host plant therefore has influence on the microorganisms colonising it, which means that different plant species growing next to each other may be colonised by different microbiomes [72]. Samad et al. [73] evaluated the microbiome compositions of grape and some weed species growing in the same field, using the 16S rRNA gene; their findings are consistent with our research and suggest that the different species hits different microbiomes. Plants that are distantly related phylogenetically show greater variation in microbiome composition, suggesting a role of plant phylogeny in the formation of microorganisms [72]. The results of the statistical analysis indicate that *Delftia* bacteria isolated from cultivated plants can be suitable components of biopreparations with anti-pathogenic effects due to the positive correlation with the activity of hydrolytic enzymes. Moreover, bacteria isolated from weeds show a promising potential in mitigation abiotic stresses due to the positive correlation and high activity of ACC deaminases.

Comparison of the results presented in this manuscript with previously published findings [26] confirms the choice of the strains ES2 (*Stenotrophomonas maltophilia*) and ZS2 (*Delftia acidovorans*) as potential effective components of multi-task biopreparations (biostimulation, protection, and phytoremediation). These strains showed high metabolic activity in the Biolog GEN III test, a high efficiency of N_2_ fixation and the ability to synthase indole-3-acetic acid (IAA) and siderophores [26]. Our previous study [26] showed that these isolates were resistant to low salinity (1%, 4%, 8%) and pH (5 and 6) levels, which indicates that they can be successfully used in non-optimal environmental conditions to stimulate plant growth and protect plants against abiotic stress at the same time. Interestingly, these strains are also characterised by a high ability to synthesise siderophores as well as iron-complexing compounds. These features may be particularly important under conditions of abiotic stress caused by metal contamination. In addition, the high activity of PGP of strain ES2 is confirmed by the clear distinction of this strain in the PCA, both in our current and previous studies [26].

In conclusion, amongst the tested strains of endophytic bacteria, a diverse ability to produce compounds positively affecting the growth and development of plants was observed. The evaluated strains were characterised by a wide range of mechanisms increasing stress tolerance in plants: lowering ethylene levels by producing ACC deaminase, degrading the cell wall of pathogens through the synthesis of hydrolytic enzymes and competing with pathogens through the production of iron-complexing compounds. An important aspect of this study is the identification of a relatively high number of bacteria that have a wide range of plant hosts (e.g., *Delftia* spp.). Therefore, these bacteria can be the core of endophytic bacteria in a given area (core of native bacteria) and may have so-called “endophytic competences” related to the effectiveness of establishing interactions with plants. Our current and previous studies provide valuable information for the selection of the most promising endophytic bacteria for plant growth and protection. Strains ES2 (*Stenotrophomonas maltophilia*) and ZS2 (*Delftia acidovorans*), characterised by direct mechanisms of plant growth promotion and some of the highest deaminase and protease activities, showed the ability to produce ICC and TPC and use sugars, amino acids, and carboxylic acids. The strains of endophytic bacteria characterised here show potential for further research on the practical use as components of a multi-task biopreparation (protection, biostimulation, and phytoremediation), which is in line with the assumptions of sustainable agriculture and environmental protection. In our future studies, the selected strains will be further evaluated in greenhouse and field experiments, in natural soil and under ambient climate conditions.

## Data Availability

All data generated or analysed during this study are included in this published article.
